# Typhoid in India: An Age-old Problem With an Existing Solution

**DOI:** 10.1093/infdis/jiab441

**Published:** 2021-11-23

**Authors:** Supriya Kumar, Raj Shankar Ghosh, Harish Iyer, Arindam Ray, Kirsten Vannice, Calman MacLennan, Tanya Shewchuk, Duncan Steele

**Affiliations:** 1 Enteric and Diarrheal Diseases, Bill & Melinda Gates Foundation, Seattle, Washington, USA; 2 India Country Office, Bill & Melinda Gates Foundation, New Delhi, India; 3 Global Delivery Program, Bill & Melinda Gates Foundation, Seattle, Washington, USA

**Keywords:** typhoid, India, TCV, cost of illness

## Abstract

Enteric fever continues to impact millions of people who lack adequate access to clean water and sanitation. The typhoid and paratyphoid fever burden in South Asia is broadly acknowledged, but current estimates of incidence, severity, and cost of illness from India are lacking. This supplement addresses this gap in our knowledge, presenting findings from two years of surveillance, conducted at multiple sites between October 2017 and February 2020, in the Surveillance for Enteric Fever in India (SEFI) network. Results provide contemporaneous evidence of high disease burden and cost of illness—the latter borne largely by patients in the absence of universal healthcare coverage in India. Against a backdrop of immediate priorities in the COVID-19 pandemic, these data are a reminder that typhoid, though often forgotten, remains a public health problem in India. Typhoid conjugate vaccines, produced by multiple Indian manufacturers, and recommended for use in high burden settings, ensure that the tools to tackle typhoid are an immediately available solution to this public health problem.

## TYPHOID IN INDIA

Typhoid is an age-old disease that was recognized in the 1880s to be caused by the bacterium *Salmonella enterica* serovar Typhi [1, 2]. It was largely eliminated from high-income countries with the development of drinking water and sanitation infrastructure and centralized water treatment [3, 4] but remains prevalent in populations without adequate access to clean water and sanitation.

According to Global Burden of Disease estimates, more than half of the 14.3 million global cases of enteric fever in 2017 occurred in India, where it is estimated that 8.3 million cases and 72 000 deaths occurred [5]. Multicountry surveillance efforts, such as the Diseases of the Most Impoverished Program, found that typhoid incidence between 2003 and 2004 was 214/100 000 among all ages in the Indian site of Kolkata [6, 7]. An earlier study in Delhi found that incidence was 980/100 000 in people younger than 40 years [8]. A recent review of the literature suggested that there remains a continued burden of disease in India, if reducing slowly over time [9], possibly due to gains in water and sanitation. Current, age-specific burden estimates are necessary to inform evidence-based policy to tackle this disease.

To fill this gap in evidence, the Christian Medical College, Vellore, set up the Surveillance for Enteric Fever in India (SEFI) network of enteric fever surveillance sites across the country with funding from the Bill & Melinda Gates Foundation [10]. Active, community-based surveillance to estimate enteric fever incidence in 4 pediatric cohorts was complemented with hospital-based hybrid surveillance for severe enteric fever at 6 hospitals, and laboratory-based surveillance for antimicrobial resistance in *S.* Typhi and *S.* Paratyphi A isolates at 9 laboratories across the country [11].

This supplement includes findings from 2 years of surveillance, conducted between October 2017 and February 2020 at various SEFI sites, with reports on incidence, case fatality, cost of illness, antimicrobial resistance, and cost-effectiveness of vaccination strategies. The reports highlight a continued high burden of enteric fever as evidenced from a typhoid incidence rate of 610/100 000 from community-based surveillance in a pediatric cohort in Delhi [12], and a case-fatality ratio (CFR) of 0.73% among hospitalized cases [13]. This overall CFR, however, masks the very high fatality among enteric fever complications caused by ileal perforation, estimated to be 7.1% in SEFI [14]. In a recent meta-analysis of case fatality rates, the median typhoid intestinal perforation-associated CFR in Asia across studies was 1% (ranging from 0% to 8.4%), with an overall CFR among 999 intestinal perforation cases of 4.6% [15]. This further highlights the importance of the SEFI network in providing a current estimate of typhoid CFR and underscoring the continued danger of severe typhoid in India.

SEFI also provides a vital update to the picture of antimicrobial resistance among *S.* Typhi isolates from India. The study reports that only 2% of 2373 isolates were multidrug resistant [16], continuing a declining trend in multidrug resistance rates reported earlier [17]. The fluoroquinolone resistance rate remains high, confirming the low utility of these drugs for typhoid treatment in India. Whereas cephalosporin resistance was not found in SEFI isolates, azithromycin resistance was observed in 1 isolate, and isolates from North India had higher minimum inhibitory concentrations than those from South India. In conjunction with recent reports of azithromycin resistance in isolates from Chandigarh [18], and in travelers from India [19], it is important to closely monitor resistance to this drug, which along with amoxicillin and cephalosporin is often prescribed for febrile illnesses in the community [20]. Rates of extensively drug resistant typhoid have been rising in Pakistan [21, 22], and azithromycin resistance has been observed in multiple neighboring countries [23, 24]. Investing in vaccines to prevent typhoid in India could preempt the rise of *S.* Typhi strains resistant to currently prescribed antibiotics. Furthermore, genomic analyses of isolates from SEFI, along with isolates from neighboring countries, could also shed light on the bacterial lineages being transmitted in the South Asian region, and whether particular parts of the region are at risk of either introduction of lineages of concern, or the emergence of resistant lineages due to migration or antibiotic use patterns. Genomic analyses can also reveal whether antibiotic resistance determinants are chromosomal rather than plasmid derived (which has implications for reversal to susceptibility).

Another important contribution of the SEFI network has been to measure the cost of hospitalization due to severe enteric fever [25] to patients and caregivers. Previous estimates of cost of illness were from a single study in Delhi in 2004, and did not include nonmedical and indirect costs [26]. As Kumar et al report in this supplement, nonmedical and indirect costs represent 50% and 37% of total costs in secondary and tertiary care centers, respectively, showing that patients and caregivers bear a substantial financial burden over and above paying for health care [27]. Most patients used savings or salary to pay for the cost of health care—public health insurance did not cover any care in secondary care centers, and only 19% of patients in tertiary care centers received assistance from insurance schemes. The finding that 1 in 5 patients borrowed or sold assets to pay for care highlights the urgent need for policies to prevent enteric fever in families in India and provide respite to those most in need.

Further evidence that the most vulnerable remain at risk of enteric fever comes from a case-control study nested within community-based surveillance in Vellore, India. Giri et al report that parents eating street-vended foods and not treating drinking water were risk factors for typhoid in children [28]. Furthermore, 14% of the household contacts of children with confirmed typhoid were found to be shedding *S.* Typhi in their stool [29], suggesting that entire households are at risk of infection and onward transmission. Better water and sanitation in households could reduce the burden of typhoid, but access to these remains limited in an urban setting like Vellore.

The SEFI national surveillance network has been an opportunity to use established methods, such as health care utilization surveys as part of hybrid surveillance, in different parts of the country. Raju et al [30] document variation in health care utilization for febrile illness across sites in India. The network has also provided the opportunity to develop novel methods for enteric fever surveillance. Raghava et al report results from a pilot typhoid environmental surveillance study in Vellore. Low-cost methods for typhoid surveillance are necessary to estimate national and subnational typhoid incidence to inform TCV use and impact given the lack of availability of blood culture, and the low sensitivity of this method in younger children and those with mild illness [31].

Against the backdrop of the SARS-CoV-2 pandemic, age-old diseases such as typhoid often fade from official priority even as the problem remains in the background. Enteric fever is a disease that drifts in incidence and antimicrobial resistance patterns, but also in policy prioritization when more urgent health issues arise. Whereas typhoid in 2021 may not be the global pandemic priority that coronavirus disease 2019 (COVID-19) is, SEFI findings have shown that it continues to impact people, especially the poorest and most vulnerable. We have effective preventive tools available, indicating it is a problem that can be addressed.

## SHORT-TERM SOLUTIONS ARE AVAILABLE

Internationally licensed vaccines against typhoid have been available for over 25 years, and although endorsed by the World Health Organization (WHO) in 2000 [32], and reaffirmed in 2008 [33], have not been widely introduced. Parenteral Vi-polysaccharide (Vi-PS) vaccines and an oral, live-attenuated vaccine, Ty21a, were shown to be safe and efficacious in preventing clinical disease [33]. Vi-PS, prequalified by WHO in 2011 [34], is licensed as a single-dose vaccine down to 2 years of age, but protection rapidly wanes from about 70% [35] to 50% by 3 years postvaccination [36]. Although the Vi-PS and Ty21a vaccines were preferable over first-generation reactogenic inactivated whole-cell vaccines [32], reasons reported for limited typhoid vaccine introduction include insufficient data on disease burden and high-risk groups to support national decision making, lack of a financing mechanism, lack of proven efficacy below 2 years of age, waning protection with time, challenging implementation strategies given the recommended schedules of the available typhoid vaccines (eg, 3 doses of Ty21a for primary immunization) [37], and hesitancy while waiting for the availability of typhoid conjugate vaccines.

Interestingly, the Vi-PS vaccine has been routinely used in Delhi since 2004, based on local perception of high disease burden. Rongsen-Chandola et al [12] report in this supplement, however, that there is limited coverage with a single dose of the typhoid Vi-PS vaccine, routinely administered to children at a median age of 30 months. Although this may have led to a reduction in typhoid burden among children younger than 5 years compared to earlier estimates of incidence [8], burden remains high in children up to 15 years of age.

With the development and licensure of many successful conjugate vaccines, including against *Haemophilus influenzae* type B, and several *Streptococcal pneumoniae* serotypes and meningococcus serogroups, typhoid Vi conjugate vaccines were seen as the solution to the limitations of other vaccine platforms. In 2008, recognizing that the available typhoid vaccines were insufficient to meet country needs, Gavi, the Vaccine Alliance (Gavi) board approved financing for typhoid conjugate vaccines (TCV), which were still under development [38]. This represents the first time the Gavi board committed to supporting a vaccine still under development. It would take another 10 years for a TCV—manufactured by Bharat Biotech in India—to be prequalified by WHO [39], and thus eligible for UN procurement and Gavi cofinancing.

## TYPHOID CONJUGATE VACCINES

Proof of concept for efficacy in children of a TCV was first demonstrated 20 years ago with a vaccine consisting of Vi-PS conjugated to recombinant *Pseudomonas aeruginosa* exoprotein A (Vi-rEPA) [40]. In Vietnamese children aged 2 to 5 years, efficacy against typhoid was 91% after 27 months, falling only marginally to 89% after 46 months [41]. India led the way in the development of the first commercial TCVs with the Drug Controller General of India, Ministry of Health and Family Welfare approving 3 Vi-tetanus toxoid vaccines [42] developed by Bio-Med, Ltd in 2008 (PedaTyph) [43], Bharat Biotech India, Ltd in 2013 (Typbar-TCV) [44], and Zydus Cadila, India (ZyVAC TCV) in 2018 [45]. A Vi-CRM_197_ vaccine developed by Biological E, Ltd, India, in 2020 (TYPHIBEV), was recently approved based on safety and immunogenicity [46].

Two of these licensed TCVs have now been prequalified by WHO, establishing their availability for procurement by Gavi and UNICEF for use in countries with high disease burden globally. Typbar TCV was prequalified in 2018, supported by data from a typhoid controlled human infection model study in Oxford [47], which showed that Typbar TCV had an efficacy of 87.1% against clinically relevant typhoid fever (fever ≥ 38.0°C and bacteremia) [47]. In 2021, TYPHIBEV, with similar levels of immunogenicity as Typbar TCV, was the second TCV to be prequalified by the WHO. In India, where typhoid vaccines are available on the private market, it is estimated that 550 000–600 000 doses are administered per year (market value of approximately US $14 million) (personal communication, Pharmarack). Randomized controlled trials and public sector introductions of Typbar TCV, including in India, are providing data and experience with the use and utility of this vaccine to support decision making on TCV introduction through public sector investments.

## TCV EFFECTIVENESS IN NAVI MUMBAI

The first public sector introduction of TCV was in Navi Mumbai, Maharashtra. Assisted by the WHO and US Centers for Disease Control and Prevention (CDC), the Navi Mumbai Municipal Corporation (NMMC) introduced TCV in 2018 among 9-month-old to 14-year-old children in the city [48]. The introduction was conducted as a demonstration project aimed at estimating campaign introduction costs, safety, effectiveness, and impact of the vaccine.

Addressing the recommendations from WHO Strategic Advisory Group of Experts on Immunization to collect safety data on Typbar TCV during introductions [49], Stanford University, WHO, and CDC researchers worked with NMMC staff to collect data on adverse events following immunization (AEFI) and hospitalized adverse events of special interest (AESI) among vaccinated individuals. Longley et al recently reported that no AEFIs or AESIs were related to the vaccine [50]. These data from the introduction of TCV among 113 420 children in Navi Mumbai add to the growing and robust safety data on this vaccine arising from trials in Nepal [51], Malawi [52], and Burkina Faso [53].

Date et al have also reported an effectiveness estimate of 80.2% (95% confidence interval [CI], 53.2%–91.6%) for Typbar TCV from a case-control study in Navi Mumbai [54]. These data add to a very consistent picture of efficacy of the vaccine from the controlled human infection model study [47] and subsequent field efficacy studies in Asia and Africa. For example, in Nepal, 12-month vaccine efficacy was 81.6% (95% CI, 58.8%–91.8%; *P* < .001) against *S*. Typhi bacteremia in children aged 9 months to 16 years [51].

With multiple TCVs licensed in India, vaccination against typhoid could have a major impact in driving down burden in India, but is also likely to be costly. Ryckman et al [55] provide a cost-effectiveness analysis of various TCV introduction strategies based on rural, urban, and age-specific modeled disease estimates for each state in the country [56]. Results suggest that routine introduction of TCV into the childhood immunization schedule at 9 months of age in rural and urban areas, alongside either national or urban one-time campaigns to immunize children up to 15 years of age would reduce burden over a 10-year time horizon and, importantly, be cost saving in India compared to the status quo. Support for the substantial costs of campaigns from Gavi are expected to be an important consideration and may ease decision making among policy makers weighing India’s many health and vaccine priorities. Introduction of TCV into the national immunization schedule will promote equity in typhoid control in India by ensuring access to the vaccine among those unable to buy TCV on the private market, where it is already available.

## CONCLUSION

India has a strong, robust process and history for the introduction of new vaccines based on evidence of the burden of illness and the cost effectiveness to the country. Recent introductions of locally produced rotavirus vaccines, for example, were built on the strong evidence of rotavirus burden emerging from surveillance data in the country, cost-effectiveness analyses, and vaccine immunogenicity and efficacy. Similar examples exist for pneumococcal vaccines and HPV where reliable national data provided the pathway to new vaccine introduction.

These vaccines are all universal in the sense that every child should receive the vaccine—for typhoid fever this is different. As the data presented in this supplement show, burden is present in the most vulnerable communities in India, those without access to safe and treated water and improved sanitation. The incidence rates in young children are amongst the highest seen globally, representing another vulnerable group that would benefit from nationally sanctioned TCV campaigns. Based on the high effectiveness shown in a local study in Navi Mumbai, the availability of multiple TCVs in India and the consistent protection seen in populations outside India (including Nepal, Bangladesh, and Pakistan), a decision by the National Technical Advisory Group for Immunization and the Ministry and Health would address an important health issue in India. Importantly, the cost effectiveness, including a cost-saving perspective seen in some analyses, makes this a rational decision for the country. Finally, investing in vaccines to prevent typhoid in India could curtail the rise of *S.* Typhi strains resistant to currently prescribed antibiotics, and further devastation caused by this disease.

## Notes

***Potential conflicts of interest.*** C.M. declared that he is a named patent inventor on *Salmonella* conjugate vaccines patent #US20160263213A1. The author has not and does not receive any payments/royalties from this patent which is now owned by GSK. All authors: No reported conflicts of interest. All authors have submitted the ICMJE Form for Disclosure of Potential Conflicts of Interest. Conflicts that the editors consider relevant to the content of the manuscript have been disclosed.

***Supplement sponsorship.*** This supplement is sponsored by the Christian Medical College Vellore Association.
